# The Brief Solastalgia Scale: A Psychometric Evaluation and Revision

**DOI:** 10.1007/s10393-024-01673-y

**Published:** 2024-03-05

**Authors:** Bruce K. Christensen, Conal Monaghan, Samantha K. Stanley, Iain Walker, Zoe Leviston, Emily Macleod, Rachael M. Rodney, Lisa-Marie Greenwood, Timothy Heffernan, Olivia Evans, Stewart Sutherland, Julia Reynolds, Alison L. Calear, Tim Kurz, Jo Lane

**Affiliations:** 1 School of Medicine and Psychology, Australian National University, Canberra, Australia; 2 Melbourne Centre for Behaviour Change, University of Melbourne, Melbourne, Australia; 3 School of Arts and Humanities, Edith Cowan University, Joondalup, Australia; 4 Centre for Entrepreneurial Agri-Technology, Australian National University, Canberra, Australia; 5 School of Built Environment, University of New South Wales, Kensington, Australia; 6 Centre for Mental Health Research, Australian National University, Canberra, Australia; 7 School of Psychological Science, University of Western Australia, Perth, Australia; 8 National Centre for Epidemiology and Population Health, Australian National University, Canberra, Australia

**Keywords:** Bushfire, Solastalgia, Environmental Distress Scale, Psychometric, Factor analysis, Short form

## Abstract

Witnessing degradation and loss to one’s home environment can cause the negative emotional experience of solastalgia. We review the psychometric properties of the 9-item Solastalgia subscale from the Environmental Distress Scale (Higginbotham et al. (EcoHealth 3:245–254, 2006)). Using data collected from three large, independent, adult samples (*N* = 4229), who were surveyed soon after the 2019/20 Australian bushfires, factor analyses confirmed the scale’s unidimensionality, while analyses derived from Item Response Theory highlighted the poor psychometric performance and redundant content of specific items. Consequently, we recommend a short-form scale consisting of five items. This Brief Solastalgia Scale (BSS) yielded excellent model fit and internal consistency in both the initial and cross-validation samples. The BSS and its parent version provide very similar patterns of associations with demographic, health, life satisfaction, climate emotion, and nature connectedness variables. Finally, multi-group confirmatory factor analysis demonstrated comparable construct architecture (i.e. configural, metric, and scalar invariance) across validation samples, gender categories, and age. As individuals and communities increasingly confront and cope with climate change and its consequences, understanding related emotional impacts is crucial. The BSS promises to aid researchers, decision makers, and practitioners to understand and support those affected by negative environmental change.

**Supplementary Information:**

The online version contains supplementary material available at 10.1007/s10393-024-01673-y.

A growing body of research examines the emotional impact of climate change and ecological disasters (e.g. Clayton & Karaszia, 2020; Hogg et al., 2021; Stanley et al., 2021). Solastalgia is one response and is characterised by sadness, grief, and powerlessness caused by the transformation and degradation of one’s environment (for a review, see Galway et al., 2019). Solastalgia has been likened to a sense of “homesickness one gets when one is still at ‘home’” (Albrecht, 2005, p. 45).

Albrecht and colleagues developed the construct through their work with residents of the Upper Hunter Region of New South Wales, Australia, who experienced significant distress living near open cut coal mines (Albrecht, 2005; Albrecht et al., 2007; Connor et al., 2004). Similar case studies identify solastalgia amongst interviews with women in the Torres Strait talking about climate change (McNamara & Westoby, 2011), farmers in rural Australia experiencing mental health effects from drought (Satore et al., 2008), and Inuit people in Canada describing the effects of the changing climate on their lives and mental health (Cunsolo et al., 2012). While most research on solastalgia has been qualitative in nature (Galway et al., 2019), Higginbotham et al. (2006) developed the Environmental Distress Scale (EDS), with a subscale measuring solastalgia. Developed from the content of interviews with the Upper Hunter community and grounded in Environmental Stress and Risk Theory, the EDS includes six subscales, namely: perceptions of environmental hazard (including both frequency and observation of hazard events), appraisal of threat, felt impact of environmental change, solastalgia, and environmental action. Higginbotham and colleagues (2006) found that solastalgia—and overall EDS scores—were higher amongst residents of the Upper Hunter compared to those in a farming community living at a distance from the environmental degradation of the open cut mine. In this study, the solastalgia subscale returned excellent internal consistency estimates (*α* = 0.93) and acceptable test–retest reliability scores (intra-class correlation = 0.73).

Researchers generally describe the EDS solastalgia subscale (EDS-S) as effective (Eisenman et al., 2015; Elser et al., 2020; Khan et al., 2012; Phillips & Murphy, 2021) and have used this measure to show that those living near degraded landscapes experience higher levels of self-reported solastalgia. For example, the EDS-S has revealed that Texans living in areas with more oil and gas wells experience heightened solastalgia (Elser et al., 2010), that most residents living through rapid urbanisation in Pakistan experienced solastalgia (Khan et al., 2012), and that solastalgia in a community in Ireland affected by coastal erosion was highest amongst long-term residents (Phillips & Murphy, 2021). In another context, Eisenman et al. (2015) observed solastalgia amongst those affected by wildfires in Arizona and that greater experiences of solastalgia predicted more severe psychological distress one year after the fires. Therefore, not only does environmental degradation appear to contribute to the experience of solastalgia, but it may also place people at risk of poorer psychological wellbeing in the future.

The consistent performance of the EDS-S notwithstanding, psychometric validation of this subscale remains limited. Although previous studies have demonstrated adequate internal consistency, with Conbrach’s alpha values ranging from 0.75 (Warsini et al, 2014) to 0.93 (Higgenbotham et al., 2006), the scale developers did not conduct a principal components or factor analysis of the solastalgia items themselves—instead verifying that the mean scores of each EDS subscale loaded onto a single factor indexing overall environmental distress. Others have variously interpreted the EDS-S as unidimensional (Eisenman et al., 2015; Luce, 2021) or multidimensional (Warsini et al., 2014) and, thus, further investigation of the underlying dimensionality is needed.

Furthermore, it is difficult to draw comparisons between past studies because each employed slightly different versions of the EDS-S items. Higginbotham et al. (2006) validated the EDS within a mining context, although they recommended that researchers adapt the measure to appraise environmental distress in the face of other environmental and human challenges, including natural disasters and human conflict/war. Subsequent users of the scale have varied the items to refer to the specific environmental disaster of interest. For example, the item “I am saddened by unwelcomed change I see in my landscape” was adjusted to “I feel sad when I look at the *landscapes damaged by the Wallow Fire*” in Eisenman et al.’s (2015) research following a wildfire, to “I feel saddened by the *loss of the beach at Courtown*” in Phillips and Murphy’s (2021) study on coastal erosion, and to “I am saddened when I look at *degraded landscapes and open-cut mine voids*” in Elser et al.’s (2020) research on the impacts of mining.

Other minor changes to the emotional response of environmental change have also been reported. For example, the item “I am ‘upset’ at the way this area looks now” has been variously replaced with ‘ashamed’ (Elser et al., 2020) or ‘disappointed’ (Phillips & Murphy, 2021; Warsini et al., 2014). Additionally, authors may select only a subset of the nine original items, either based on the items they felt were relevant (e.g. four items were used in Khan et al., 2012), or for unstated reasons (e.g. seven items were used in Phillips & Murphy, 2021). These adaptations make it unclear whether studies are capturing a similar experience of solastalgia across contexts, raising significant challenges regarding how best to compare experiences of solastalgia across environmental events (Supplementary Material A shows each iteration of the scale items that we have identified in the literature). For the purposes of the current study, wording for each item from the initial EDS-S was used. Although bespoke item wording may increase the relevance of individual items to a given context, such variability makes psychometric comparisons difficult and, therefore, the current study endorses consistent wording to bolster comparability and measurement rigour.

Collectively, these results and observations underscore the need for more focussed investigation of the dimensionality, validity, and reliability of the solastalgia subscale, which is the primary goal of the current research. We also consider solastalgia’s associations with demographic variables (age, gender, income, education, and perceived social status) and with the theoretically-relevant constructs of perceived environmental change, nature connectedness, climate emotions, identification as an environmentalist, and life satisfaction. We expected those who believe their environment is substantially worsening and who are more connected to nature and/or identify as environmentalists are likely more sensitive to environmental change and, thus, may experience higher self-reported levels of solastalgia. Higher levels of solastalgia should also relate to greater emotional responses to climate change. Lastly, and given the small associations with wellbeing in past literature (Eisenman et al., 2015), we expected solastalgia to be negatively related to life satisfaction, and positively related to ecologically related anxiety and stress. Based on Albrecht’s (2005) speculation that solastalgia can escalate into physical symptoms, we also investigated whether higher levels of solastalgia related to poorer self-rated physical health.

## Method

We analyse data from three samples that included the EDS-S. As these data were each collected for different primary purposes, there are small differences in survey methods, which are highlight below.

### Participants and procedure

Sample 1 was collected between March and April of 2020. The sample included 1,776 participants aged between 18 and 87 years (*M* = 49.80, SD = 16.51, median = 51 years; 59.10% female, 40.51% male, 0.23% ‘other’, 0.17% preferring not to say). Participants were recruited from the Canberra and surrounding regions as part of a study designed to examine the effects of the 2019–2020 bushfires on the health and wellbeing of residents affected by heavy smoke (Rodney et al., 2021), using postal invitations, a Qualtrics panel sample, and convenience sampling methods (social media and radio advertising, and local media stories). Participants were eligible for inclusion in the study if they were living in Canberra or surrounding regions between 15 December 2019 and 15 February 2020 during the 2019–2020 bushfire season. Although 2,095 completed the survey, participants were excluded if they were out of the area of interest or did not provide a valid postcode (*n* = 11), did not respond to all EDS-S items (*n* = 276), or if they were identified as multivariate outliers on EDS-S items (Mahalanobis, *χ*^2^(9) = 27.88, *p* < 0.001; *n* = 32), to reach our final sample of 1776.

Sample 2 was collected between August 20 and September 20 of 2020. The sample included 1,651 participants aged between 18 and 85 years (*M* = 44.89, SD = 17.62; median = 43 years; 46.94% female, 52.63% male, 0.18% ‘other’, 0.24% preferring not to say). Participants were recruited for a national survey that was designed to examine Australians’ attitudes, emotions, and behaviours related to climate change. Participants were recruited nationwide via a Qualtrics panel sample, with quotas in place so that the final sample was representative of the adult Australian population (based on the 2016 census) in age, gender, and location. Participants were eligible for inclusion in the national survey if the quota for their demographic group was not yet full, and if they met quality checks (i.e., passed attention checks presented in the first third of the survey, did not take less than half the median time to complete the full survey, or respond with a pattern or unrealistic responses such as unintelligible text in open-ended text boxes). In total, 5,110 participants met these quality requirements, but only those who responded to the question: “Do you think that over the last few years the quality of your local environment is getting better, staying the same, or getting worse” with response options ‘getting a bit worse’ or ‘getting much worse’ (*n* = 1,749) were presented with the EDS-S items. Of this subset of participants, we further excluded those who did not respond to all items (*n* = 30) or were identified as a multivariate outlier (significant Mahalanobis distances, *p* < 0.001; *n* = 68), to reach our final sample of 1,651.

Sample 3 was collected between January and June 2021. The sample included 802 participants aged between 18 and 86 years (*M* = 42.59, SD = 16.26, median = 41 years; 43.02% female; 56.23% male, 0.50% ‘other’, 0.25% prefer not to say). Participants were recruited as part of a nationwide survey designed to investigate impacts of Australia's 2019/2020 bushfires on mental health and wellbeing, using postal invitations, a Qualtrics paid sample, and convenience sampling methods (social media and radio advertising, and local media stories). Participants were eligible for inclusion in the larger study if they were 18-years or over, had been living in Australia since the 2019–2020 bushfire season, and passed the quality checks outlined for Sample 2. In total, 3,083 participants met these criteria, and those who reported living in a bushfire-affected postcode (identified by the Australian Government) at the start of the bushfire season (*n* = 569) or being involved in fighting the fires (*n* = 343) were deemed bushfire-affected and thus presented with the EDS-S. Of this group, multivariate outliers (*n* = 18) and those who responded with “does not apply” to any item, were removed prior to analysis, to reach a final sample of 802.

### Analytic Strategy

Data were analysed in RStudio (2022.02.3; *R* version 4.1.1). Sample 1 data were randomly split into exploratory (*n* = 855) and confirmatory (*n* = 921) subsamples. Exploratory analyses subjected the nine items from the Solastalgia scale to a schedule of item-level property analysis, with each approach providing important and complementary information about item performance. Exploratory Factor Analysis (EFA), utilising Cattell’s elbow test and parallel analysis (95th percentile, 2000 simulated samples), was used to reduce the likelihood of overdimensionalisation (van der Eijk & Rose, 2015) and to identify inadequately loading items (< 0.50 was utilised given the focus on a robust final scale). Item Response Theory (IRT; Graded Response Model [GRM]; Samejima, 1969) then estimated where (known as the location parameter) on the solastalgia continuum items provided information about participants. We focussed on balancing information while ensuring measurement across the entire − 2.5 to + 2.5 logit range (interpreted similarly to *Z*-scores). Poorer items were iteratively removed during the IRT stage based on distributional (e.g. censored / truncated or bimodal distributions) and IRT parameters. In particular, items were retained if they yielded low to moderate estimates of local dependence, robust information curves, and unique positional information on the solastalgia continuum (see Results below).

Confirmatory Factor Analysis (CFA) on the confirmatory subsample evaluated the factor structure’s replicability and structural validity (i.e., that conclusions from the exploratory subsample were not measurement artefacts or Type I errors). The robustness of the structure was then further tested through a series of CFA measurement invariance analyses (sample, age, and gender identity) across all three samples. Finally, the brief scale was subjected to external validity analysis to provide evidence for its construct validity.

## Results

### Item Reduction

An investigation of item distributions suggested that several items had skewed distributions: specifically, items 1 (positive), 2 (negative), and 3 (negative). Only item 1’s skew was considered incorrigible (censored), with all other items having appropriate variation in relation to their mean, thereby allowing adequate sampling variability. (Item distributions can be seen in Supplementary Material B.) All items were subjected to a polychloric EFA (given that the 5-point response options were not considered adequate for underlying continuous data). Data were considered appropriate for EFA based on the Kaiser–Meyer–Olkin test (0.91), and significant Barlett’s test, *χ*^2^(36) = 12,531.64, *p* < 0.001. The Scree plot and parallel analysis strongly supported a single dominant factor, with parallel analysis allowing for a very weak secondary factor. Since the Eigenvalue of the second factor was well below 1 (i.e. 0.38), the single factor solution was adopted, with item loadings ranging from 0.46 (item 1) to 0.89 (item 8). (Item loadings are available in Supplementary Material C.) At this stage, Item 1 was removed from further analyses, given the strength of the loadings of all other items (> 0.68) and the incorrigible skew identified during the previous item-analysis.

An initial round of IRT was conducted on the eight candidate items. Approximately 98% of participants fitted the model based on Zh values > − 1.96, and non-fitting participants were removed from further IRT analyses (*n* = 36) to reduce the introduction of unnecessary error variance. The assumption of local dependence (LD) was also evaluated; however, these values can be difficult to interpret with a small number of items. Four pairs of items showed low to moderate LD estimates ( >|.30|), and potentially inflated information parameters were considered when selecting the final items (Yen, 1984).

Three items that yielded substantially higher information curves than the remaining items were retained (see Supplementary Material D for the original item response curves). One additional item provided moderate information; however, because this was primarily situated below − 1.5 logits (non-solastalgia end of the continuum) it was removed. Two items provided unique information above 1.5 logits (solastalgia end of the continuum), and we chose to retain one of these items to ensure the final set contained sufficient content breadth. We named the final 5-item scale the Brief Solastalgia Scale (BSS) (Table 1; Appendix 1), and then subjected it to confirmatory analyses.

**Table 1:** Mean (Standard Deviation) Responses to Solastalgia Items Across Samples.

Item	Sample 1	Sample 2	Sample 3
1. My sense of belonging to this place has been undermined by recent changes*	2.21(1.18)	4.00(1.37)	3.00(1.61)
2. I am sad that familiar aspects of this place are disappearing (e.g. animals, plants, landmarks, open space)	4.00(1.02)	5.43(1.28)	3.98(1.63)
3. I am worried that aspects of this area that I value are being lost	3.73(1.08)	5.28(1.25)	3.90(1.58)
4. I miss having the peaceful feeling that I once enjoyed by being in this place	3.16(1.22)	4.89(1.38)	3.77(1.63)
5. I am upset at the way this area looks now	2.98(1.14)	4.63(1.47)	3.63(1.62)
6. My lifestyle is being threatened by change in my local area	2.74(1.19)	4.16(1.47)	3.46(1.58)
7. Unique aspects of nature that made this place special are being lost forever	3.37(1.20)	4.90(1.38)	3.80(1.60)
8. I am saddened by unwelcome change I see in my landscape	3.42(1.14)	4.96(1.33)	3.79(1.58)
9. I feel powerless to stop unwanted changes to this place	3.33(1.13)	5.03(1.38)	–
Mean solastalgia score (of 9 items)	3.22 (.85)	4.81(1.04)	3.67(1.31)
Mean solastalgia score (of 5 items)	3.24 (.94)	4.79(1.14)	3.72(1.37)
Response scale	1–5	1–7	1–5

Item 1 varied slightly by sample (see Method section for details). Bolded items are those retained in the Brief Solastalgia Scale.

Item 9 not included in Sample 3. Standard deviation in brackets.

### Final Scale Evaluation and Confirmatory Analyses

We evaluated measurement invariance, sequentially constraining parameters across each group to equality. This places a higher standard of equivalence between the models in each sample. After initially fitting the model to all samples without constraints (configural invariance), we sequentially constrained the factor loadings (metric), intercepts (scalar), and then means (strict) across each group to equality. A substantial reduction in model fit, ΔCFI > 0.01, suggests these parameters are not equivalent between the groups (Table 2).

**Table 2:** CFA Fit Statistics, Internal Consistency, and Fidelity for the Solastalgia Factor in Each Sample.

Sample	Test Statistic		Incremental Fit		Absolute Fit			Internal Consistency		Correlation with 9-item scale
	*χ*^2^	*p*	CFI	NNFI	SRMR	RMSEA	RMSEA 90% CI	Alpha	Omega	Pearson
1a								.89	.89	.97
1b	2.719	.743	1.000	1.002	.015	.000	[.000, .033]	.88	.89	.97
2	9.716	.084	.999	.997	.025	.024	[.000, .046]	.89	.89	.97
3	7.779	.169	.999	.998	.027	.026	[.000, .060]	.91	.91	.98

Sample 1a is the exploratory subsample, and Sample 1b is the confirmatory subsample. Model 1 has all five items loading onto a single.

solastalgia factor, models estimated using Diagonally weighted Least Squares (DWLS). Baseline *df* = 10, and model *df* = 5. Maximum likelihood estimation also returned similar fit estimates (Hu & Bentler, 1999) (see Supplementary Material D).

Three series of invariance analyses were conducted, comparing samples, binary gender identities, and then across age cohort. As age is a continuous variable, we created three age groups, < -1SD, -1SD to 1SD, and > 1SD. Across all comparisons, the solastalgia scale was invariant at the configural, metric, and scalar levels (Chen, 2007; Cheung & Rensvold, 2002), indicating that these factor structures are equivalent without any systematic bias that might skew mean level comparisons (scalar invariance) (Table 3).

**Table 3:** CFA invariance fit statistics for the Solastalgia factor across samples and across age groups.

Analysis	Model	Test Statistic			Alternative Fit	
		^2^	*df*	*p*	CFI	RMSEA
Sample	Configural	23.504	15	.974	.999	.020
	Metric	35.900	23	.042	.999	.020
	Scalar	133.421	31	< .001	.992	.049
Binary Gender Identity	Configural	16.550	10	.85	1.000	.018
	Metric	21.905	14	.081	.999	.016
	Scalar	35.594	18	.008	.999	.022
Age	Configural	18.326	15	.246	1.000	.013
	Metric	26.277	23	.288	1.000	.010
	Scalar	38.599	31	.164	.999	.013

Model invariance tested without estimated residual covariance for parsimony. All models estimated using Diagonally weighted Least Squares (DWLS). Unfortunately, only binary gender identities were included in the analyses due to sample size constraints.

**Figure 1:**
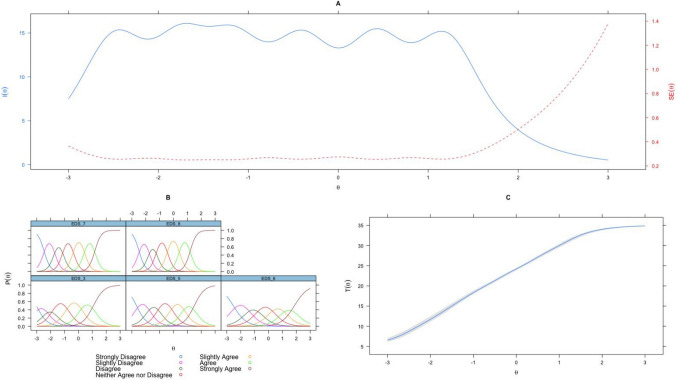
Item information curves and item response category curves for the nine items of the EDS-Solastalgia scale.

**Figure 2:**
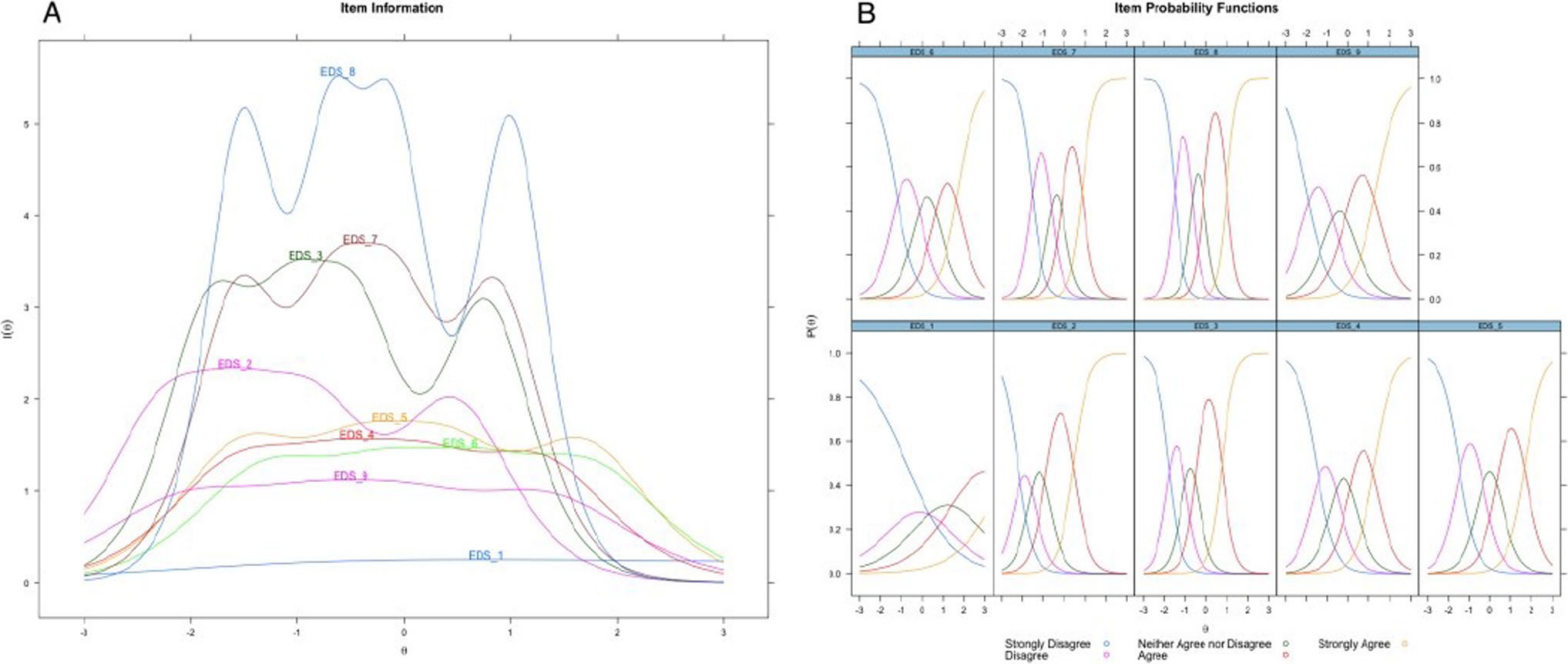
**A** Information curves for each of the 9 Solastalgia items. **B** Item probability functions for each of the 9 Solastalgia items. *Note*. A higher resolution version will be attached upon publication.

Finally, IRT analyses were run on the final scale (Fig. 1) using Sample 2 to maximise the sample size of participants not already used in scale development and to ensure the robustness of the IRT parameters across samples and response scales. The results demonstrated that all items provided strong information about the middle of the latent trait, with relatively clear distinctions between each of the response category information curves (information ranged from 2.13 to 4.41; See Supplementary Material D.II for IRT parameters). In addition, the final scale captured the majority of its information in the − 2 to 2 logit range, ideal for studies on the general population (Fig. 2).

### External Validity

External validity analysis correlated BSS scores with external variable scores in Sample 2 (Table 4). Demographic groups experienced solastalgia at similar levels, though solastalgia is experienced to a slightly lower degree amongst those who tend to perceive themselves as higher in subjective social status. Solastalgia is also related to lower life satisfaction, though unrelated to subjective health. We also found that people experiencing solastalgia tended to feel more connected to nature, experience more intense negative emotions about climate change, and were more likely to report that their environment is getting *much* worse than a *bit* worse.

**Table 4:** Correlations between the Solastalgia scale and external validity variables.

Domain	Measure	Brief Solastalgia scale
Demographics	Age	− .04
	Gender^+^	.03
	Income	.02
	Education	.02
	Subjective social status	− .07**
Wellbeing	Life satisfaction	− .10***
	Subjective health	− .03
Environmental	Nature relatedness	.42***
	Eco-anxiety	.32***
	Eco-depression	.31***
	Eco-anger	.31***
	Identification as an environmentalist	.29***
	+ Local environmental change (1 = getting much worse, 2 = getting a bit worse)	− .26***

Results are from Sample 2. Correlations with binary variables are point-biserial correlations.

^*^p < .05, ** *p *< .01, *** *p* < .001, ^+^Binary variable given sample size.

## Discussion

This is the first study to systematically evaluate solastalgia measurement through factor analytic and item response approaches. Here, a refined 5-item BSS is presented. The BSS performed well, correlating strongly with the longer 9-item scale, with high internal consistency, and a robust single factor structure. Its outstanding psychometric performance, even with just over half the number of items of the original scale, enables expedient measurement of solastalgia to facilitate the quantitative study of distress resulting from environmental degradation. The BSS performed well regardless of whether it was operationalised using a 5-point or 7-point Likert scale.

Interestingly, exploratory and confirmatory factor analyses strongly supported a single underlying dimension of solastalgia, clarifying previous uncertainty surrounding its unidimensional (Eisenman et al., 2015) or multidimensional (Warsini et al., 2014) nature. Therefore, individuals do not appear to differentiate between core aspects of solastalgia (e.g. worry, loss, powerlessness), instead seeing each aspect contributing towards the greater negative emotional experience. Measurement invariance also indicated that this structure was consistent across ages, samples, and binary gender identities. This key finding indicates that how participants view solastalgia does not vary between bushfire (Samples 1 and 3) and more general environmental change (Sample 2). It also suggests that demographic groups view solastalgia in the same way, and older people perceive solastalgia the same way as younger people. Importantly, this invariance enables valid comparisons of mean differences across groups and samples, as there are no systematic differences in how solastalgia is measured that would artificially influence mean solastalgia scores.

Experiences of solastalgia have, to date, predominantly been understood through interviews (Galway et al., 2019). Such qualitative work is informative as it provides a rich understanding of the lived experience of environmental distress. However, quantitative approaches are needed to learn about the prevalence of solastalgia and how intensely it is experienced across groups and in relation to other environmental attitudes. It is also useful for researchers and community organisations, such as those focussed mental health and environmental advocacy, to have at their disposal a very brief and economical indicator of solastalgia. Contributing to these gaps, the BSS reveals associations comparable to the full 9-item scale with related constructs and supports the idea that solastalgia is felt more intensely by those who perceive greater environmental change, by those who are more connected to nature, and those who experience more intense emotions when thinking about climate change. Although Albrecht and colleagues (2005, 2007) have suggested that solastalgia may be a ‘psychoterratic illness’ capable of manifesting in physical illness, we did not find an association with self-rated health. This was consistent when solastalgia was operationalised using the EDS-Solastalgia and BSS and, thus, reflects that solastalgia can be experienced independent of general physical wellbeing.

Expressions of concern and grief about changes to one’s landscape are key characteristics of solastalgia (Albrecht, 2007). Supporting the face validity of the BSS, the items we retained captured worry and sadness about environmental change and loss and how this threatens one's lifestyle, suggesting these are the core components of solastalgia. However, people experiencing environmental change also express feelings of powerlessness and a disrupted sense of belonging (Albrecht, 2007; Phillips & Murphy, 2021). Interestingly, items tapping these experiences were not retained in the BSS because they were less informative than items capturing lifestyle change—i.e. a sense of loss and a sense of sadness due to environmental change. This may suggest that feelings of belonging and powerlessness are context specific, or related to (but separate from) solastalgia itself.

While the BSS demonstrates many desirable psychometric characteristics, several limitations of scale and the current study should be acknowledged. Standardised error increases rapidly outside approximately 95% of the middle of the latent trait (− 2 to 2 logit range), despite specifically selecting items to capture the higher ends of solastalgia through IRT. As a result, this BSS is ideal for measuring the general response (population) to solastalgia. To measure the high ends, new item/s specifically designed for this population would be useful (there were no candidate items in the original 9 items that met this criterion). Potentially, those heavily affected may have unique and traumatic stories that are more appropriate for interview or short answer responses rather than brief self-report instruments. Additionally, whether the BSS is equally valid in measuring solastalgia across varied environmental alterations (e.g. floods, bushfires, mining, deforestation) cannot be adjudicated by the current data and requires additional research to address this issue. Similarly, the current results do not yet support the use of the BSS as a clinical or individual measure of solastalgia since normative data were not collected across a representative population. Instead, the samples here represent individuals at-risk for solastalgia given their recent experience of the 2019/20 Australian bushfires and/or their experience of local environmental decline. To estimate quantitative departures from normal levels of solastalgia, an appropriate normative sample would have to be ascertained and could be a constructive focus for future research. In the meantime, the level of solastalgia in the current sample can be estimated from the aggregated mean, and standard deviation, across the three samples for the BSS, which is 3.19 (0.87).

## Conclusions

Our work builds on Higginbotham and colleagues’ (2006) substantive scale development efforts by producing a valid and reliable 5-item solastalgia scale. Importantly, and supported by measurement invariance, we present the BSS as a standard set of items. While the original authors recommend altering the items to the population and environmental degradation of interest, we recommend retaining the current item wording to reduce item heterogeneity and increase the validity of cross-study and cross-sample comparisons. This means that solastalgia scores can be compared more generally to understand the distress evoked by different environmental events, and monitor how solastalgia changes across time, circumstances and communities, which is particularly relevant as the climate crisis continues.

### Electronic supplementary material

Below is the link to the electronic supplementary material.

Supplementary file1 (DOCX 370 KB)

## Appendix 1: The brief Solastalgia scale

### Instructions

Please rate (circle the number that best describes) the extent you agree or disagree with the following statements relating to change in your local environment.

### Scoring Instructions

Calculate the mean score based on responses to all survey items. Please note, there are no reversed-scored items.

### Statements

1. I am worried that aspects of this area that I value are being lost.

2. I am upset at the way this area looks now.

3. My lifestyle is being threatened by change in my local area.

4. Unique aspects of nature that made this place special are being lost forever.

5. I am saddened by unwelcome change I see in my landscape.

## References

[CR1] Adler NE, Epel E, Castellazzo G, Ickovics J (2000). Relationship of subjective and objective social status with psychological and physiological functioning: preliminary data in healthy white women. Health Psychology.

[CR2] Albrecht G, Sartore GM, Connor L, Higginbotham N, Freeman S, Kelly B, Pollard G (2007). Solastalgia: the distress caused by environmental change. Australasian Psychiatry.

[CR3] Albrecht G (2005). “Solastalgia”. A new concept in health and identity. PAN Philosophy Activism Nature.

[CR4] Chen FF (2007). Sensitivity of goodness of fit indexes to lack of measurement invariance. Structural Equation Modelling.

[CR5] Cheung GW, Rensvold RB (2002). Evaluating goodness-of-fit indexes for testing measurement invariance. Structural Equation Modelling: A Multidisciplinary Journal.

[CR6] Clayton S, Karazsia BT (2020). Development and validation of a measure of climate change anxiety. Journal of Environmental Psychology.

[CR7] Connor L, Albrecht G, Higginbotham N, Freeman S, Smith W (2004). Environmental change and human health in upper hunter communities of new south Wales Australia. EcoHealth..

[CR8] Cunsolo Willox A, Harper SL, Ford JD, Landman K, Houle K, Edge VL (2012). “From this place and of this place:” climate change, sense of place, and health in Nunatsiavut Canada. Social Science & Medicine.

[CR9] Eisenman D, McCaffrey S, Donatello I, Marshal G (2015). An ecosystems and vulnerable populations perspective on solastalgia and psychological distress after a wildfire. EcoHealth.

[CR10] Elser H, Goldman-Mellor S, Morello-Frosch R, Deziel NC, Ranjbar K, Casey JA (2020). Petro-riskscapes and environmental distress in West Texas: community perceptions of environmental degradation, threats, and loss. Energy Research & Social Science.

[CR11] Galway LP, Beery T, Jones-Casey K, Tasala K (2019). Mapping the solastalgia literature: a scoping review study. International Journal of Environmental Research and Public Health.

[CR12] Higginbotham N, Connor L, Albrecht G, Freeman S, Agho K (2006). Validation of an environmental distress scale. EcoHealth.

[CR13] Hogg TL, Stanley SK, O’Brien LV, Wilson MS, Watsford CR (2021). The hogg eco-anxiety scale: development and validation of a multidimensional scale. Global Environmental Change.

[CR14] Hu LT, Bentler PM (1999). Cutoff criteria for fit indexes in covariance structure analysis: conventional criteria versus new alternatives. Structural Equation Modelling: A Multidisciplinary Journal.

[CR15] Khan NY, Ghafoor N, Iftikhar R, Malik M (2012). Urban annoyances and mental health in the city of Lahore Pakistan. Journal of Urban Affairs.

[CR16] Luce, C. (2021), Grief, loss and climate change: validation of a solastalgia scale. [Doctoral dissertation thesis, Virginia Commonwealth University].

[CR17] McNamara KE, Westoby R (2011). Solastalgia and the gendered nature of climate change: an example from Erub Island. Torres Strait. Ecohealth.

[CR18] Nisbet EK, Zelenski JM (2013). The NR-6: A new brief measure of nature relatedness. Frontiers in Psychology.

[CR19] Phillips C, Murphy C (2021). Solastalgia, place attachment and disruption: insights from a coastal community on the front line. Regional Environmental Change.

[CR20] Rodney RM, Swaminathan A, Calear AL, Christensen BK, Lal A, Leviston Z, Reynolds J, Trevenar S, Vardoulakis S, Walker I (2021). Physical and mental health effectos fo bushfire and smoke in the Australian capital territory 2019–20. Frontiers of Public Health.

[CR21] Samejima F (1969). Estimation of latent ability using a response pattern of graded scores. Psychometrika Monograph Supplement.

[CR22] Sartore GM, Kelly B, Stain H, Albrecht G, Higginbotham N (2008). Control, uncertainty, and expectations for the future: a qualitative study of the impact of drought on a rural Australian community. Rural and Remote Health.

[CR23] Stanley SK, Hogg TL, Leviston Z, Walker I (2021). From anger to action: differential impacts of eco-anxiety, eco-depression, and eco-anger on climate action and wellbeing. The Journal of Climate Change and Health.

[CR24] van der Eijk C, Rose J (2015). Risky business: Factor analysis of survey data—assessing the probability of incorrect dimensionalisation. PLoS ONE.

[CR25] Warsini S, Buettner P, Mills J, West C, Usher K (2014). Translation, cultural adaptation, and psychometric testing of the environmental distress scale withIndonesian survivors of a volcanic eruption. Disaster Medicine and Public Health Preparedness.

[CR26] Yen WM (1984). Effects of local item dependence on the fit and equating performance of the three-parameter logistic model. Applied Psychological Measurement.

