# Supplementary material for: The Brief Solastalgia Scale: A Psychometric Evaluation and Revision
Source: Ecohealth. 2024 Mar 5;21(1):83–93. doi: 10.1007/s10393-024-01673-y (PMC11127848; doi:10.1007/s10393-024-01673-y)
Supplement: Supplementary file 1 — Supplementary file1 (DOCX 370 KB) [file 10393_2024_1673_MOESM1_ESM.docx]

**Supplementary Material**

**Supplementary Material A – Review of Variation in Solastalgia Scale Items Across Studies**

|  | **Latest solastalgia items from Higginbotham (personal communication, July 6, 2019)** | **Higginbotham et al. (2006)** | **Eisenman et al. (2015)** | **Elser et al. (2020)** | **Khan et al. (2012)** | **Phillips & Murphy (2021)** | **Warsini et al. (2014)** |
| --- | --- | --- | --- | --- | --- | --- | --- |
|  | Open cut mine | Open cut mine | Wildfire | Oil and gas wells | Rapid urbanisation | Coastal erosion | Volcanic eruption |
| 1 | My sense of belonging to this place has been undermined by unwelcome change. | Sense of belonging undermined by change |  | My sense of belonging to this place has been undermined by unwelcome environmental change. | Sense of belonging undermined by the environmental change | My sense of belonging to this place has been undermined by the loss of the beach. | My sense of belonging to this place has been undermined by unwelcome change |
| 2 | I am sad that familiar aspects of this place are disappearing (e.g., animals, plants, landmarks, open space). | Sad that familiar animals and plants are disappearing |  | I am sad that familiar animals, plants, and fish are disappearing from this place. |  | Sometimes I find myself thinking about times when the beach was larger | Feel saddened to look at degraded landscapes, and everything that is buried |
| 3 | I am worried that aspects of this area that I value are being lost. | Worried that valued aspects of place—clean air and water, scenery—are being lost | I feel that aspects of living near the forest that I value were lost after the Wallow Fire | I am worried that aspects of this place that I value are being lost. e.g. Clean air and water, beautiful scenery |  | I am worried that the valued aspects of Courtown are being lost | Feel worried that the valued aspects of this place are being lost |
| 4 | I miss having the peaceful feeling that I once enjoyed by being in this place. | Miss peace and quiet once enjoyed in this place | Seeing the forest affected by the Wallow Fire has been stressful | I miss having the sense of peace and quiet once enjoyed in this place. | Missing peace once enjoyed |  | Miss having the sense of peace and quiet I once enjoyed in this place |
| 5 | I am upset at the way this area looks now. | Ashamed of the way this area looks now | I feel like I have been grieving for the loss of the forest affected by the Wallow Fire | I am ashamed of the way this area looks now. | Feeling ashamed the way this area looks | I feel disappointed in the way Courtown looks now | Feel disappointed in the way this area looks now |
| 6 | My lifestyle is being threatened by change in my local area. | Farming lifestyle depending on good land and water is threatened by change | I have gone to the area affected by the Wallow Fire less than I did before the fire | A farming lifestyle that depends on good land and water is being threatened by environmental change. |  | I feel that the beach suited my way of life more in the past | A farming lifestyle is being threatened by environmental change |
| 7 | Unique aspects of nature that made this place special are being lost forever. | Unique aspects of nature in this place are being lost | Unique aspects of nature in this place were lost after the Wallow Fire | Unique aspects of nature that made this place special are being lost forever. |  | I am upset over the loss of the beach because it is part of my heritage | Feel good about the restoration of the environment (eg, rehabilitation) |
| 8 | I am saddened by unwelcomed change I see in my landscape. | Sad when look at degraded landscapes and mine voids | I feel sad when I look at the landscapes damaged by the Wallow Fire | I am saddened when I look at degraded landscapes and open cut mine voids. | Feeling sad looking at degraded environment | I feel saddened by the loss of the beach at Courtown | Feel sad about current situation |
| 9 | I feel powerless to stop unwanted changes to this place. | Thought of my family being forced to leave this place upsets me |  | I am worried that my family may be forced to leave this place. |  |  | The thought of government forcing me to leave this place is upsetting |

**Supplementary Material B – Item Distributions for all 9 Solastalgia Items in Sample 1A**

**
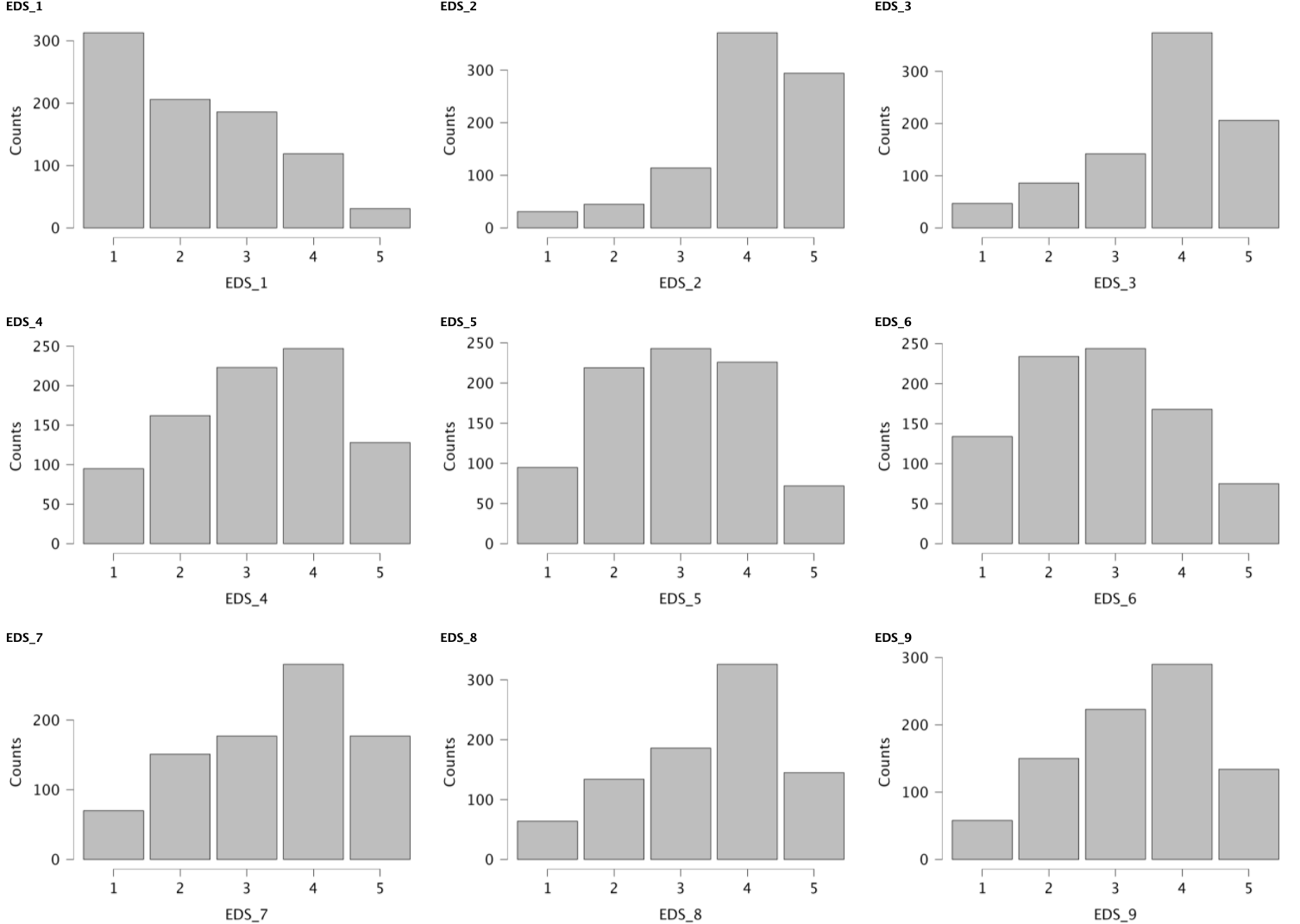
**

**Supplementary Material C – Polychloric EFA Estimates**

| Item | Loading | Communalities | Uniqueness |
| --- | --- | --- | --- |
| 1 | .46 | .22 | .78 |
| 2 | .81 | .65 | .35 |
| 3 | .88 | .77 | .23 |
| 4 | .79 | .62 | .38 |
| 5 | .76 | .58 | .42 |
| 6 | .78 | .60 | .40 |
| 7 | .85 | .73 | .27 |
| 8 | .89 | .79 | .21 |
| 9 | .69 | .47 | .53 |

**Supplementary Material D – Item Response Theory**

**D.I: Item Response Curves for the 9 Item Solastalgia Scale**


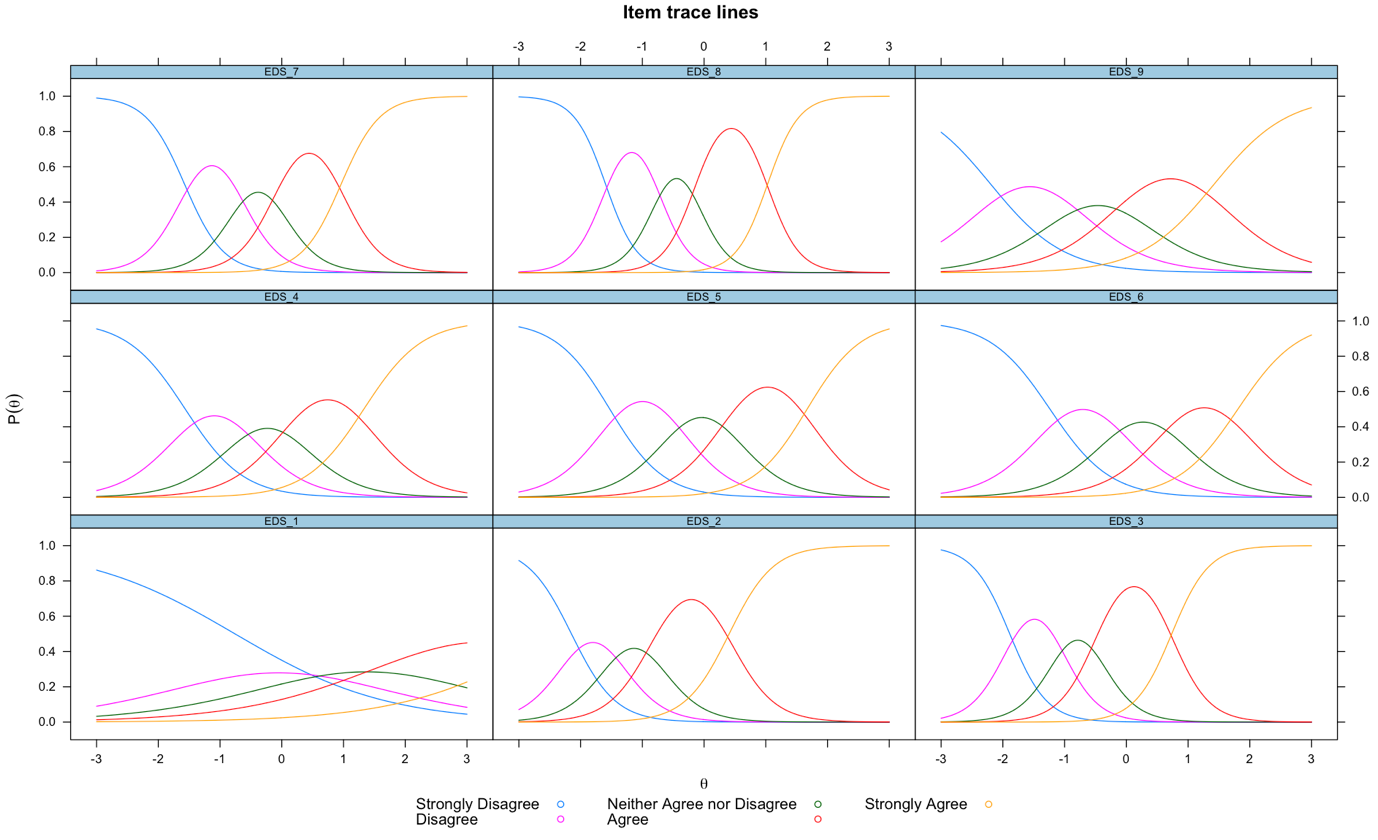


**D.II: Final IRT Parameters for the 5-Item Solastalgia Scale**

|  | a | b1 | b2 | b3 | b4 |
| --- | --- | --- | --- | --- | --- |
| EDS_3 | 2.964 | -2.029 | -1.136 | -0.512 | 0.818 |
| EDS_4 | 2.403 | -1.545 | -0.595 | 0.123 | 1.325 |
| EDS_5 | 2.956 | -1.437 | -0.482 | 0.356 | 1.529 |
| EDS_6 | 2.285 | -1.16 | -0.112 | 0.734 | 1.844 |
| EDS_8 | 3.627 | -1.657 | -0.78 | -0.16 | 1.166 |

*N = 884. CFA sample without Zh outliers*
